# To evaluate the analgesic effectiveness of bilateral erector spinae plane block versus thoracic epidural analgesia in open cardiac surgeries approached through midline sternotomy

**DOI:** 10.1186/s44158-024-00148-4

**Published:** 2024-03-01

**Authors:** Hilal Ahmad Bhat, Talib Khan, Arun Puri, Jatin Narula, Altaf Hussain Mir, Shaqul Qamar Wani, Hakeem Zubair Ashraf, Suhail Sidiq, Saima Kabir

**Affiliations:** 1 Department of Anaesthesiology, Sher I Kashmir Institute of Medical Sciences (SKIMS), Srinagar, Jammu and Kashmir 190011 India; 2 Division of CardioVascular & Thoracic Anaesthesia and Cardiothoracic Surgical Intensive Care Unit, Sher I Kashmir Institute of Medical Sciences (SKIMS), Srinagar, Jammu and Kashmir 190011 India; 3 Department of Anaesthesiology and Pain Management, Max Super-Specialty Hospital Patparganj, New Delhi, 110091 India; 4 Department of Cardiac Anaesthesia, Amrita Hospital, Faridabad, Haryana 121002 India; 5 Department of Radiation Oncology, Sher I Kashmir Institute of Medical Sciences (SKIMS), Srinagar, Jammu and Kashmir 190011 India; 6 Department of Cardiovascular and Thoracic Surgery, Sher I Kashmir Institute of Medical Sciences (SKIMS), Jammu and Kashmir, Srinagar, 190011 India; 7 Department of Critical Care Medicine, Sher I Kashmir Institute of Medical Sciences (SKIMS), Srinagar, Jammu and Kashmir 190011 India

**Keywords:** Erector spinae plane block, Thoracic epidural analgesia, Open cardiac surgeries, Median sternotomy, Perioperative analgesia, Enhanced recovery after cardiac surgery, Rescue analgesia, Visual analogue scale

## Abstract

**Background:**

The efficacy of the erector spinae plane (ESP) block in mitigating postoperative pain has been shown for a range of thoracic and abdominal procedures. However, there is a paucity of literature investigating its impact on postoperative analgesia as well as its influence on weaning and subsequent recovery in comparison to thoracic epidural analgesia (TEA) in median sternotomy-based approach for open-cardiac surgeries and hence the study.

**Methods:**

Irrespective of gender or age, 74 adult patients scheduled to undergo open cardiac surgery were enrolled and randomly allocated into two groups: the Group TEA (thoracic epidural block) and the Group ESP (bilateral Erector Spinae Plane block). The following variables were analysed prospectively and compared among the groups with regard to pain control, as determined by the VAS Scale both at rest (VAS_R_) and during spirometry (VAS_S_), time to extubation, quantity and frequency of rescue analgesia delivered, day of first ambulation, length of stay in the intensive care unit (ICU), and any adverse cardiac events (ACE), respiratory events (ARE), or other events, if pertinent.

**Results:**

Clinical and demographic variables were similar in both groups. Both groups had overall good pain control, as determined by the VAS scale both at rest (VAS_R_) and with spirometry (VAS_S_) with Group ESP demonstrating superior pain regulation compared to Group TEA during the post-extubation period at 6, 9, and 12 h, respectively (*P* > 0.05). Although statistically insignificant, the postoperative mean rescue analgesic doses utilised in both groups were comparable, but there was a higher frequency requirement in Group TEA. The hemodynamic and respiratory profiles were comparable, except for a few arrhythmias in Group TEA. With comparable results, early recovery, fast-track extubation, and intensive care unit (ICU) stay were achieved.

**Conclusions:**

The ESP block has been found to have optimal analgesic effects during open cardiac surgery, resulting in a decreased need for additional analgesic doses and eliminating the possibility of a coagulation emergency. Consequently, it presents itself as a safer alternative to the potentially invasive thoracic epidural analgesia (TEA).

## Introduction

Median sternotomy in cardiac surgery if not optimally managed causes significant acute postoperative pain, and can lead to impairment in pulmonary functions with increased morbidity and mortality. Optimal analgesia not only improves patient comfort but allows early ventilator weaning with lowering of respiratory complications. A myriad of analgesic techniques have been employed to mitigate this post-surgical pain. Opioids though the commonly used analgesia, are associated with delay in tracheal extubation and recovery of bowel and bladder function in the postoperative period in addition to sedation, respiratory depression, nausea and vomiting [1, 2]. Evidence evaluation is necessary for comparing the advantages of various pain management approaches, guiding clinical practice, and identifying topics for additional research.

Following cardiac surgery, thoracic epidural analgesia (TEA) can offer excellent opioid-free analgesia and is linked to decreased rates of respiratory problems, arrhythmias [3], and mortality [4]. However, due to the risk of epidural haemorrhage, its usage in cardiac surgery is still restricted. A new fascial plane block called the erector spinae plane (ESP) block places local anaesthetic (LA) in a myofascial plane between the erector spinae muscle and the thoracic transverse processes [5–7]. The ESP block has a favourable risk–benefit profile since the site of injection is distant from the neuraxis, major blood vessels, pleura, and may be considered even in the presence of coagulation abnormalities [8]. The utilisation of bilateral and ipsilateral erector spinae plane (ESP) blocks has proven to be efficacious analgesia in cardiac surgical procedures performed using median sternotomy and thoracotomy approaches, respectively [8–10].

Keeping in view the various concerns of TEA in cardiac surgeries, we undertook the study to evaluate the feasibility and effectiveness of ESP block over TEA in cardiac surgeries and compare the two with respect to various factors intraoperatively and postoperatively.

## Materials and methods

### Settings and design

**Fig. 1:**
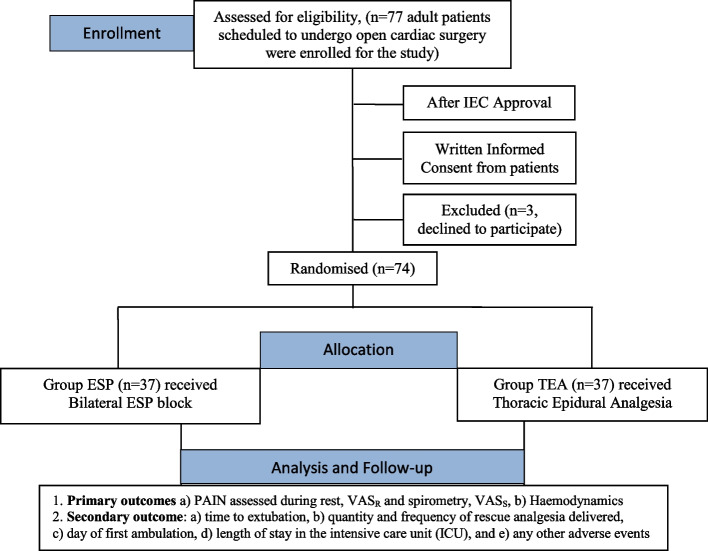
CONSORT flow diagram depicting study design. (IEC = Institutional Ethics Committee; Group ESP = erector spinae plane block group; Group TEA = Thoracic epidural analgesia group; hemodynamics include HR = heart rate and MAP = mean arterial pressure; VAS_R_ = visual analogue scale at rest and VAS_S_ = visual analogue scale during spirometry)

After obtaining approval from the institutional ethics council (IEC) and written informed consent from the patients, this prospective randomized study was carried out in the Division of Cardiovascular and Thoracic Anaesthesiology and Intensive Care Unit (Department of Anaesthesiology) of a tertiary care referral and teaching hospital of Northern India (Fig. 1).

### Resources and approach

After excluding the three patients, the study included 74 adult patients scheduled for open cardiac surgery with normal to moderate left ventricular dysfunction, regardless of demographic profile, gender, age, or American Society of Anaesthesiologists (ASA) physical status. The patients were randomised using a computer-generated table of random numbers and allocated into two groups: Group ESP (those who received ultrasound-guided bilateral Erector Spinae Plane blocks) and Group TEA (those who received high thoracic epidural blocks guided by ultrasound).

### Exclusion criteria

The study excluded patients who refused to undergo surgery or regional anaesthesia, or who underwent emergency or repeat surgery, or who had CAD with main left vessel disease, severe left ventricular (LV) dysfunction (ejection fraction of less than 30%), unstable hemodynamics requiring intra-aortic balloon pumps (IABP), inotrope support and/or cardiac pacing, atrial fibrillation on anticoagulation therapy, coagulation and bleeding disorders (International normalised ratio, INR > 1.5), or hepatic dysfunction (AST and ALT of more than twice normal), renal dysfunction (as indicated by their creatinine clearance < 60 ml/min), and allergy to ropivacaine.

### Patient education and assessment

All patients scheduled for surgery had their baseline demographic information recorded. Patients were instructed, reassured, and informed to indicate the severity of their postoperative pain using the eleven-point visual analogue scale (VAS), which is rated from 0 to 10, with 0 denoting the least amount of discomfort possible and 10 as the most intense pain imaginable.

### Preoperative advise

With the exception of aspirin, anti-platelet drugs were stopped at least 5 days before surgery, and other preoperative medications were continued up until the morning of the surgery. Two hours before surgery, alprazolam 0.25 mg and pantoprazole 40 mg were given orally together with a sip of clear water. All patients received an intra-dermal test dosage of an antibiotic (tazobactam and piperacillin), and each patient's response was recorded in their medical records.

### Operating room planning

In the operating room (OR), patients were welcomed, reassured and explained the anaesthetic plan and procedure. The WHO surgical safety check was performed in all the procedures before positioning the patients on the operating table. After local anaesthetic (L/A) skin infiltration, a 16-G intravenous (IV) angiocatheter was secured preferably on the dorsum of the left hand for administration of fluids and medication. After completing the modified Allen’s test, a 20 G intra-arterial angiocatheter (BD FloSwitch TM arterial cannulae, 20 G/1.10 mm × 45 mm, Belgium) was secured in the left radial artery after L/A infiltration using point of care ultrasonography guidance (POCUS) (Fuji film Sonosite Inc.’s Sonosite Edge II Total; Transducer HFL 38, 13–6 MHz) with scan depth of 1–3 cm in all patients.

### Monitoring

Multiparametric monitoring [which includes 5-lead ECG, digital pulse oximetry (SPO2), temperature (both core *T*_c_ and surface *T*_s_) monitoring, invasive blood pressure (IBP), capnography, central venous pressure (CVP)], was carried out in accordance with the institutional protocol.

### Regional blocks

Depending on the group assigned (Group ESP or Group TEA), the relevant blocks were carried out in a seated position under all aseptic precautions after L/A infiltration at the level corresponding to the T_4_–T_5_ vertebrae by a qualified anaesthesiologist before the induction of general anaesthesia (GA). In both groups, the block was administered using a 16G Tuohy's needle (Portex Epidural minipack, System 1, Smiths Medical ASD, Inc. USA).

**Fig. 2:**
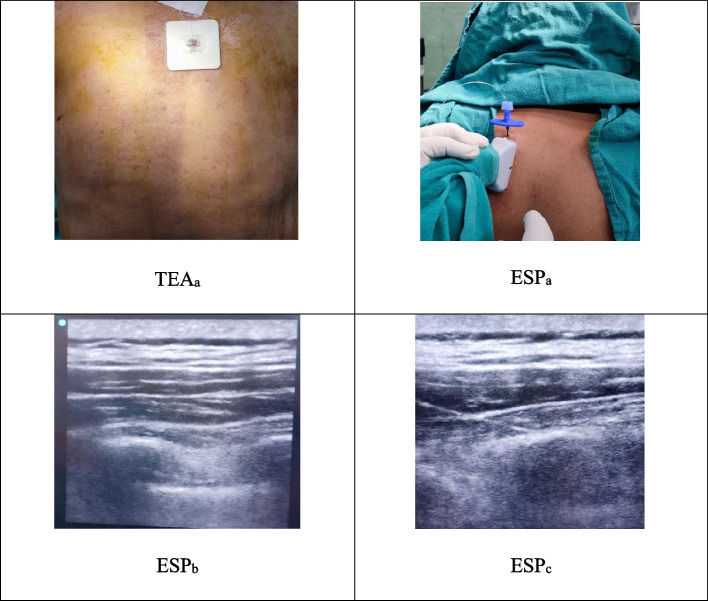
TEA_a_ = thoracic epidural in place; ESP_a_ = probe position and needle placement for ESP block; ESP_b_ = ultrasound image of the block with muscles and transverse process visible; ESP_c_ = ultrasound image of the needle with drug visible separating the erector spinae muscle from transverse process

In the Group TEA, after assessing the depth of epidural (Ep) space using POCUS, a 16G Tuohy’s needle was inserted into the Ep space confirmed by loss of resistance to saline which was followed by the insertion of an 18 G Ep catheter into the Ep space 5 cm beyond the needle tip and was fixed in place after creating a subcutaneous tunnel and secured underneath the transparent dressing. The inadvertent intravascular and intrathecal placement was ruled out using freshly prepared lignocaine 2% and adrenaline. After the induction of anaesthesia, a bolus of 10 ml of ropivacaine 0.2% was gently given in increments, and then a maintenance infusion was started at the rate of (@) 5 ml/h (Fig. 2, TEA_a_).

In the Group ESP, bilateral ESP blocks were carried out assisted by POCUS with scan depth of 3–5 cm. The probe was oriented longitudinally 2–3 cm lateral to T5 spinous process (Fig. 2, ESP_a_) and a 16-G Tuohy’s needle was inserted using the in-plane technique to the ultrasound beam in a craniocaudal direction to achieve contact with the T4 transverse process (Fig. 2, ESP_a_, ESP_b_, and ESP_c_). Correct needle tip position was confirmed by visualising the linear spread of 5–10 ml of normal saline injected that separates erector spinae muscle from the transverse process (Fig. 2, ESP_c_). An 18G catheter was inserted 5 cm beyond the needle tip under POCUS visualisation until it reached the vicinity of the T_4_ transverse process. The catheter was secured by making a subcutaneous tunnel fixed under the transparent dressing. A contralateral catheter was inserted with the same technique and secured in a similar fashion. Both the catheters were fixed aseptically and connected to two separate filters. 10 ml of ropivacaine 0.2% was injected in each catheter and was followed by continuous infusion @ 5 ml/h after induction of GA.

### Induction and maintenance of GA

GA was induced by using etomidate 0.3 mg/kg, midazolam 0.1 mg/kg, fentanyl 3 mcg/kg and rocuronium 1.0 mg/kg as a muscle relaxant for facilitating direct laryngoscopy (DL) and endotracheal tube (ETT) insertion in all the patients. ETT was secured in position after the bilateral chest auscultation for equal air entry with elastic adhesive and was connected to the anaesthesia workstation using a circular breathing circuit. The respiratory parameters were kept as per the ideal body weight with tidal volume (V_t_) of 6 ml/kg, respiratory rate (RR) of 12–16, I:E ratio of 1:2, PEEP of 5 cm of H_2_O and FiO_2_ of 40–60% (to keep PaO_2_ of 90–100 mmHg). Trans-esophageal echocardiography (TEE) probe was inserted after induction of GA for intraoperative real-time cardiac assessment. Surgery was performed in all cases by a single surgeon as per standard operative protocols.

Intraoperative hemodynamic monitoring of heart rate (HR), arterial invasive blood pressure (IBP), central venous pressure (CVP), end-tidal CO_2_ (EtCO_2_) and SpO_2_ was carried out in all the patients. Hemodynamic instability was managed with the help of continuous infusion of intravenous inotropes epinephrine, norepinephrine, and/or dobutamine as per the standard dose, titrated to keep mean arterial pressure (MAP) of 60–70 mmHg throughout the surgery. Intraoperative hypertension was treated with IV nitroglycerin as per the standard dose of 0.25 to 0.5 mcg/kg/min and titrated as per the desired MAP. IV fluids were administered in all patients as per the left ventricular volume assessment using TEE. Systemic heparinisation was reversed with protamine-sulphate 1.3 times the dose of heparin administered. Intraoperative response to pain was assessed by an increase in HR and MAP > 20% above the baseline value and was treated with IV fentanyl boluses of 1–1.5 mcg/kg in both groups.

### Post-anesthesia care

At the end of the surgery, all patients were shifted to the cardiac surgical intensive care unit (CSICU) for elective postoperative mechanical ventilation. Continuous infusion of ropivacaine 0.2% @ 3–5 ml/h was administered in both groups for postoperative analgesia via each catheter. All patients were planned for early fast-tracking extubation once the criteria for weaning and extubation were met. The postoperative pain assessment was done by utilising VAS scoring immediately after extubation until the first 72 h. Pain assessment was done every 3 hourly intervals till the first 12 h followed by 6 hourly till 48 h and thereafter 12 hourly till 72 h. All nursing staff and doctors on duty were instructed to administer routine as well as rescue analgesics strictly as per the study protocol. Breakthrough pain was controlled by IV Paracetamol 1 g q8 hourly in all the patients.

### Objective outcomes

Primary outcome measures analysed include pain using VAS scale (0–10) at rest (VAS_R_) and with spirometry (VAS_S_). Secondary outcome parameters include total perioperative opioid consumption, mean dose of rescue analgesia and their frequency, duration of mechanical ventilation, time to extubation, length of ICU stay, time to first ambulation, efforts at spirometry, incidence of arrhythmias (AF), and time to first oral intake (Table 2).

### Statistical methods

Numbers (n) and percentages (%) were used to represent categorical variables, and mean ± standard deviation (SD) and median were used to represent continuous variables. The normality of the data was assessed using the Kolmogorov–Smirnov test. In the event that the assumption of normality was rejected, a non-parametric test was employed.

Quantitative variables were compared between groups using the independent t-test/ Mann–Whitney test (where data sets were not normally distributed). Chi-square/Fisher's exact tests correlated qualitative variables. A *P* value of < 0.05 indicates statistical significance. MS Excel spreadsheet was used for data entry, while Statistical Package for Social Sciences (SPSS) version 16.0 (Chicago, Inc., USA) was used for analysis.

## Results

**Fig. 3:**
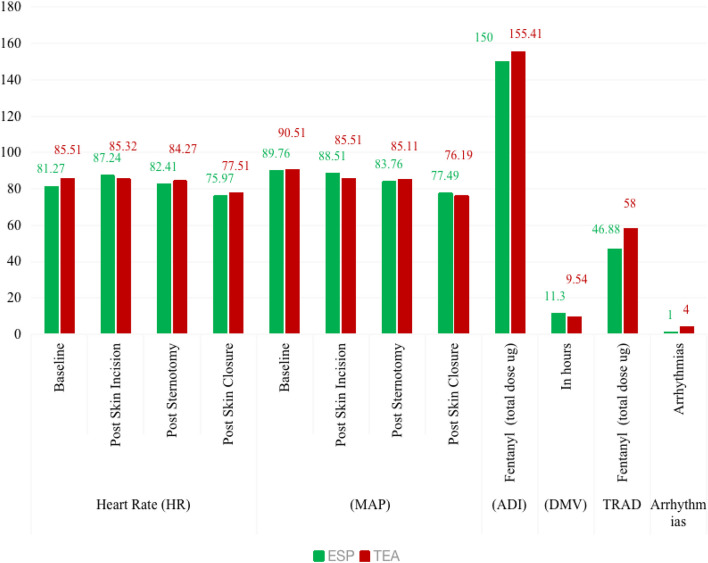
Temporal trend of perioperative haemodynamic parameters. (HR = heart rate; MAP = mean arterial pressure; ADI = analgesic dose intraoperative (fentanyl in micrograms, µg); DMV = duration of mechanical ventilation; TRAD = total rescue analgesic doses (fentanyl in µg); ESP = Group ESP; TEA = Group TEA)

The statistical analysis did not yield a significant difference between the two groups in terms of demographic data, anthropometric characteristics, preoperative clinical indicators and ASA status (Table 1). The HR (heart rate) and MAP (mean arterial pressure) were similar in both groups at various time points during the surgical procedure, including baseline, after the skin incision, after the sternotomy, and after the skin closure. The Group TEA required a slightly higher intraoperative dose of fentanyl, 155.41 ± 25.77 mcg, as compared to the Group ESP, 150.00 ± 28.86 mcg and the difference was not statistically significant (P value = 0.39) (Fig. 3).

**Table 1:** Demographic and preoperative clinical profile

Clinico-demographic parameters		ESP, (*n* = 37)	TEA, (*n* = 37)
Age in years	(Mean ± SD)	61.27 ± 12.60	59.22 ± 10.69
Gender, *n* (%)	Male	26 (70.3)	27 (73.0)
	Female	11 (29.7)	10 (27.0)
Anthropometry, mean ± SD	Height in cms	166.24 ± 8.76	164.43 ± 10.01
	Weight in kgs	69.76 ± 13.02	68.51 ± 13.27
	BMI in kg/m^2^	25.14 ± 3.74	25.18 ± 3.54
ASA, *n* (%)	II	1 (2.70%)	4 (10.81%)
	III	34 (91.89%)	30 (81.08%)
	IV	2 (5.40%)	3 (8.10%)
MPG, *n* (%)	I	2 (5.41%)	0
	II	32 (86.48%)	34 (91.89%)
	III	3 (8.11%)	3 (8.11)
Surgeries performed, *n* (%)	ASD closure	0	3 (8.10%)
	MVR	3 (8.10%)	1 (2.70%)
	AVR	2 (5.40%)	0
	CABG	30 (81.08%)	32 (86.48%)
	LA myx removal	1 (2.70%)	1 (2.70%)
	LA myx removal + CABG	1 (2.70%)	0
LVEF %	(Mean ± SD)	54.41 ± 5.77	51.05 ± 3.86

*ESP* erector spinae plane block group, *TEA* thoracic epidural analgesia group, *SD* standard deviation, *n* number, % percentage, *ASA* American Society of Anaesthesiology Physical Status, *ASD* atrial septal defect, *MVR* mitral valve repair, *AVR* aortic valve repair, *CABG* coronary artery bypass grafting, *LA myx* left atrial myxoma, *LVEF* left ventricular ejection fraction, *cms* centimetres, *kg* kilograms, *m* metres

**Fig. 4:**
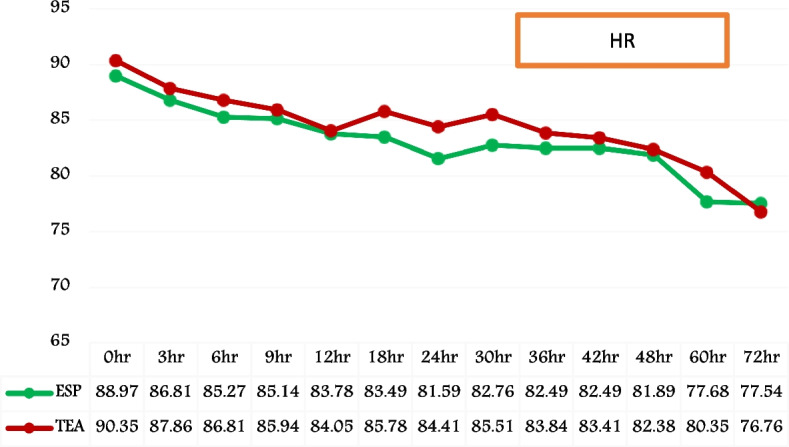
Temporal trend of post-extubation heart rate (HR). (HR = heart rate; ESP = Group ESP; TEA = Group TEA; h = post-extubation hours)

**Fig. 5:**
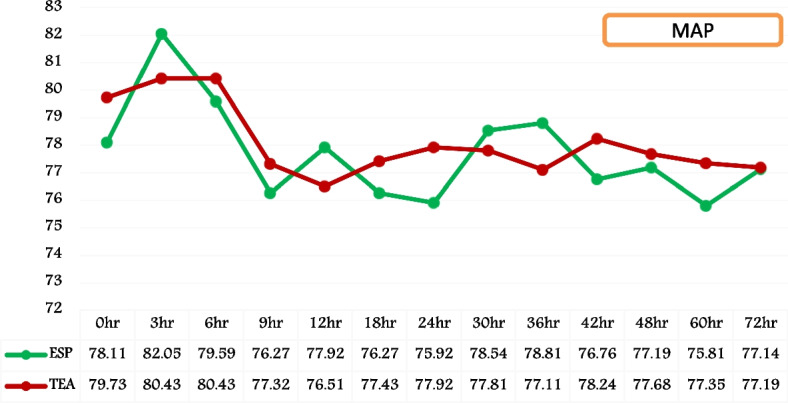
Temporal trend of post-extubation mean arterial pressures (MAP). (MAP = mean arterial pressure; ESP = Group ESP; TEA = Group TEA; h = post-extubation hours)

HR and MAP exhibited similar patterns in both groups following extubation, as evidenced by the graphical and temporal trends seen over a 72-h period. The statistical analysis yielded a non-significant P value (Figs. 4 and 5).

**Fig. 6:**
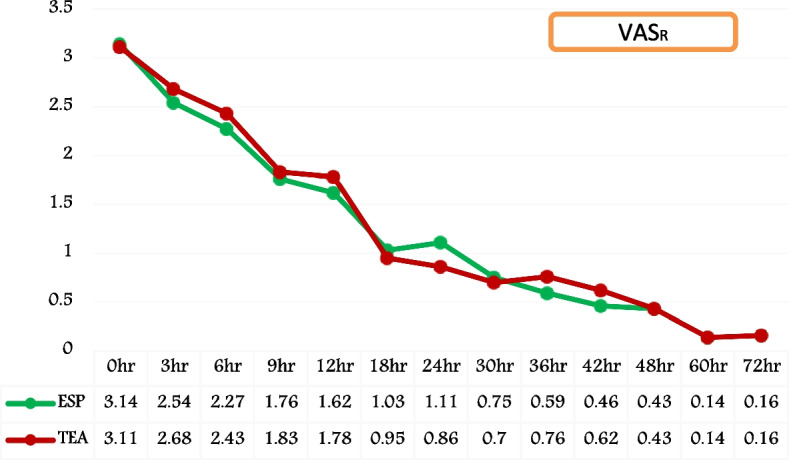
Temporal trend of post-extubation VAS score at rest (VAS_R_). (VAS_R_ = visual analogue scale at rest; ESP = Group ESP; TEA = Group TEA; h = post-extubation hours)

**Fig. 7:**
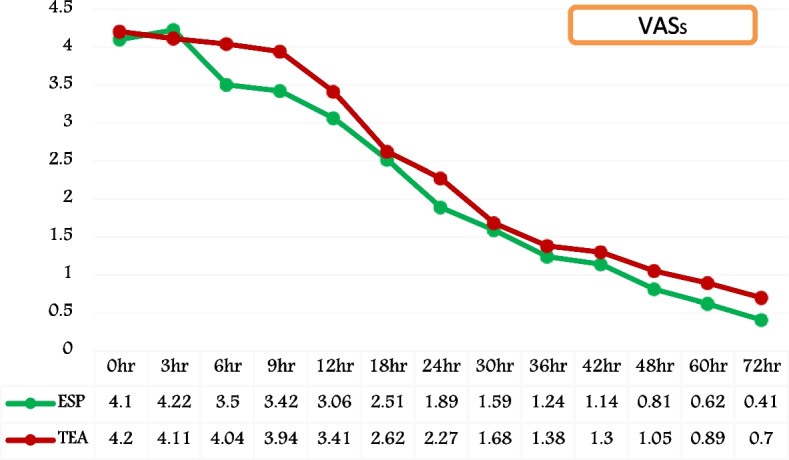
Temporal trend of post-extubation VAS score during spirometry (VAS_S_). (VAS_S_ = visual analogue scale with spirometry; ESP = Group ESP; TEA = Group TEA; h = post-extubation hours)

The VAS score at rest (VASR) exhibited comparable outcomes in both groups, indicating effective pain management within the observed 72-h period following extubation and remained at or below 4, and the statistical analysis showed no significant difference, (*P* value > 0.05) (Fig. 6). Though the difference was statistically insignificant, VAS score during spirometry (VAS_S_) has demonstrated superior analgesia with score of < 4 in Group ESP at 6 h, 9 h, and 12 h compared to score of ≥ 4 with Group TEA, (*P* value > 0.05) (Fig. 7).

During the post-extubation period, the mean doses of rescue analgesia of fentanyl were slightly higher in the Group TEA (58.00 ± 21.11 μg) as compared to the Group ESP (46.88 ± 8.83 μg) suggesting better pain control in Group ESP; however, the difference was not statistically significant (*p* value = 0.17). Furthermore, it was noted that the frequency of rescue analgesia doses administered was higher in the cohort receiving thoracic epidural analgesia (Group TEA), but this difference was not statistically significant (Table 2).

**Table 2:** Secondary outcome variables

Secondary outcome variables		ESP, (*n* = 37)	TEA, (*n* = 37)	*p* value
Total Analgesic doses intraoperatively, (mean ± SD)	Fentanyl (mcg)	150.00 ± 28.86	155.41 ± 25.77	0.39
Total postoperative rescue analgesic doses, (mean ± SD)	Fentanyl (mcg)	46.88 ± 8.83	58.00 ± 21.11	0.17
Frequency of rescue analgesic doses, *n* (%)	One	7 (87.5)	7 (46.7)	0.06
	Two	1(12.5)	8 (53.3)	
Duration of surgery, (mean ± SD)	In hours	4.89 ± 0.90	5.04 ± 0.95	0.49
Duration of mechanical ventilation, (Mean ± SD)	In hours	11.30 ± 5.77	9.54 ± 5.01	0.16
First day oral intake	*n* (%)	35 (94.6)	33 (89.2)	0.36
First day ambulation	*n* (%)	30 (81.1)	30 (81.1)	1.00
Length of ICU stay, (mean ± SD)	In days	2.65 ± 0.48	2.68 ± 0.62	0.83
Arrhythmias, *n* (%)	AF	1 (2.70)	4 (10.81)	0.16

*ESP* erector spinae plane block group, *TEA* thoracic epidural analgesia group, *SD* standard deviation,* n* number of patients, % percentage, *mcg* micrograms, *AF* atrial fibrillation

The mean duration of mechanical ventilation (DMV) from intubation in the operating room to extubation in the intensive care unit (ICU) was comparable between the two groups. Group ESP had a mean DMV of 11.30 ± 5.77 h, whereas Group TEA had a mean DMV of 9.54 ± 5.01 h (*P* value = 0.16).

The first oral intake and the first day of ambulation showed no significant difference between the two groups, (*P* value > 0.05). Length of ICU stay was also comparable between both the groups with a statistically insignificant *P* value of 0.83. The incidence of arrhythmias, such as atrial fibrillation (AF), was higher in the Group TEA as compared to the Group ESP; however, this difference was not found to be statistically significant (*p* value = 0.16) (Table 2).

As a result of our data interpretation, we inferred that adequate analgesia is important for early and enhanced recovery after cardiac surgery even when achieved with less invasive techniques with optimal haemodynamics and fewer complications, in addition to equivalent time to ICU stay, feeding, and ambulation.

## Discussion

Effective pain control and low opioid use are key to fast-track techniques [11, 12] and quicker extubation [13] in cardiac surgery patients [11, 12, 14]. This is challenging because, mostly due to the sternotomy incision, cardiac surgery causes moderate to severe early postoperative discomfort. Regional anaesthesia is necessary for a multimodal opioid-sparing regimen, and TEA is the most well-researched technique for heart surgery. TEA’s analgesic effects are eclipsed by the risk of epidural haematoma and the requirement for sufficient time to pass between epidural catheter insertion and systemic heparinisation. Thoracic paravertebral blockade (TPVB) has been suggested as a lower-risk alternative [15], although its efficacy has been questioned [16] and pneumothorax risk is a major concern [17].

POCUS-guided ESP blocks have negligible pneumothorax risk and are simpler than POCUS-guided TPVB because the target transverse process is more shallow and highly visible on ultrasound. The compressibility of the location and its distance from the neuraxis, main nerves, and blood arteries reduce the likelihood of clinically significant haemorrhage or hematoma. Even in anti-coagulated patients, the latest American Society of Regional Anaesthesia (ASRA) recommendations support the judicious use of ESP block when used wisely [18].

The purpose of the study was to investigate both the analgesic efficacy and duration of ropivacaine 0.2% on ESP block versus TEA in cardiac surgeries approached through median sternotomy. In the current study, the Group ESP noticed less opioid use and improved postoperative recovery with fewer complications reported. It was also simple to administer, even to cardiac patients using anti-platelets or anticoagulants without risk of epidural haematoma.

Leyva FM et al. [9] in a case series conducted in 2018 concluded that ESP block provided excellent postoperative analgesia in four out of five cases undergoing median sternotomy with minimal use of opioids which was similar to our study in terms of opioid requirement. Nagaraja PS et al. [19] in their study comparing TEA block and ESP block revealed comparable VAS scores at 0 h, 3 h, 6 h, and 12 h at rest and coughing (*p* value > 0.05), and comparable ventilator and ICU duration. Furthermore, TEA block had statistically significantly higher VAS score at 24 h, 36 h, and 48 h than ESP block with *p* value < 0.05 revealing better pain control with the ESP block. These results were comparable to our study results.

Neuraxial techniques have been researched widely by various authors in patients undergoing cardiac surgery [20–22] mainly motivated by the emergence of fast-tracking, which has the potential to enable prompt extubation and reduce the length of stay in the ICU, resulting in a shorter overall hospitalisation period. Although there is no statistically significant difference in perioperative morbidity and mortality between the use of neuraxial analgesia alone and its combination with general anaesthesia [23–26], previous studies have shown that there is a substantial difference in terms of time to extubation and the quality of analgesia provided [27, 28].

Bracco et al. [29] have reported a minimal postoperative complication such as myocardial dysfunction, pneumonia, acute renal failure and delirium in cardiac surgical patients who were administered TEA combined with GA compared with GA alone, along with the benefit of cost saving per person due to shorter ICU stay and hospital time. However, in cardiac surgical patients, a major concern in using neuraxial techniques is the safety of the procedure in patients on chronic dual anti-platelet therapy, intraoperative systemic anticoagulation, and cardiopulmonary bypass-induced coagulopathy. The incidence of epidural haematoma is unknown in cardiac surgery [30, 31]. However, the reported estimated risk of epidural haematoma with TEA is 1 in 12,000 and catheter-related epidural haematoma is 1 in 5493 [32].

There were no reported complications like hypotension, bradycardia, pneumothorax, haematoma formation, or other neural or vascular complications in our study. ESP block provided a relatively good analgesic alternative in cardiac surgeries with the benefit of early extubation, early ambulation and early discharge from the ICU and hospital.

## Conclusion

Our data interpretation inferred that optimal analgesia is critical for improved recovery and hemodynamics, and minimising complication. In conclusion, the erector spinae plane block offers prospective advantages over epidural analgesia in patients undergoing open cardiac surgery approached via median sternotomy. Its minimally invasive nature, lower risk of complications, and comparable analgesic efficacy make it an appealing and safer alternative by eliminating the possibility of a coagulation emergency, with the potential to improve postoperative pain management and overall patient outcome in this specific surgical scenario. Consequently, it presents itself as a safer alternative to the potentially invasive thoracic epidural analgesia. However, further studies are needed to determine its long-term efficacy and safety in cardiac surgery situations.

## Data Availability

All the data related to the study is available with HAB.
